# Does Phototherapy Affect Ductus Arteriosus Closure in Preterm Infants ≤32 Weeks of Gestation, and Can We Influence This Through Chest Shielding? Review of the Literature and a Meta-Analysis

**DOI:** 10.3390/biomedicines13102567

**Published:** 2025-10-21

**Authors:** Marta Simon, Zsuzsanna Gall, Monika Rusneac, Amalia Fagarasan, Raluca Marian, Madalina Anciuc-Crauciuc, Andreea Racean, Andrea Noemi Toth, Manuela Cucerea

**Affiliations:** 1 Pediatric IV Department, George Emil Palade University of Medicine, Pharmacy, Science and Technology of Targu Mures, 540142 Targu Mures, Romania; marta.simon@umfst.ro (M.S.); madalina.anciuc@umfst.ro (M.A.-C.); andreea.racean@umfst.ro (A.R.); andrea.toth@umfst.ro (A.N.T.); manuela.cucerea@umfst.ro (M.C.); 2 Department of Neonatology, Târgu Mureș County Emergency Clinical Hospital, 540136 Targu Mures, Romania; monika.rusneac@yahoo.com; 3 Pediatrics III Department, George Emil Palade University of Medicine, Pharmacy, Science and Technology of Targu Mures, 540142 Targu Mures, Romania; amalia.fagarasan@umfst.ro; 4 Cellular and Molecular Biology Department, George Emil Palade University of Medicine, Pharmacy, Science and Technology of Targu Mures, 540142 Targu Mures, Romania; raluca.marian@umfst.ro

**Keywords:** prematurity, patent ductus arteriosus, phototherapy, chest shielding

## Abstract

**Background**: Persistency of patent ductus arteriosus is the main cardiac condition in the preterm population born before 32 completed weeks of gestation with possible short- and long-term hemodynamic disturbances leading to vast morbidity. Jaundice is present in the majority of very preterms needing phototherapy, that also may have an influence on immature hemodynamics. The objectives of this review and meta-analysis were to find relevant evidence of whether chest shielding during phototherapy does or does not have an impact on the ductus arteriosus patency and hemodynamics. **Methods**: we reviewed the literature and performed a meta-analysis of five randomized controlled trials regarding chest shielding effect on the ductus arteriosus closure. **Results**: A total of 452 infants, with a mean gestational age of 28.04 weeks and mean birth weight of 1004.8 g were included in our meta-analysis, where we found an RR of 0.6 for developing PDA during phototherapy and chest shielding (95% CI: 0.37; 0.96. prediction interval: 0.18; 1.99) while development of hemodynamically significant PDA had RR = 0.57, within 95% CI: 0.3; 1.06, and a predictive interval between: 0.11; 2.93. **Conclusions**: Although the estimated RR may suggest a possible moderate protective role of the chest shield regarding development of PDA during phototherapy, the wideness of the predictive intervals, that include no effect, as well as the small number of eligible trials with heterogeneity between them, make the available data insufficient to evaluate the effectiveness of chest shielding during phototherapy. For more conclusive evidence there is a need for well-designed, blinded, multicenter randomized controlled trials with standardized assessment addressing to a more compact target population, knowing the large physiological differences among preterm infants of different gestational ages.

## 1. Introduction

Persistency of patent ductus arteriosus (PDA) is a common cardiac condition in the preterm population, particularly among very low and extremely low gestational age neonates (ELGANs) born before 32 completed weeks of gestation (or 28 weeks, respectively). When a PDA is hemodynamically significant, it can lead to various complications, including pulmonary vascular overload, kidney and splanchnic hypoperfusion, and alterations in cerebral blood flow [1,2,3,4]. The management of this condition remains a topic of ongoing debate due to its range of associated morbidities [5,6].

The ductus arteriosus (DA) is a crucial vascular connection in the fetus that links the main pulmonary artery to the aorta. This connection allows approximately 41% of the combined ventricular output to bypass the lungs and enter the systemic circulation. It remains open due to lower oxygen levels and the intramural production of prostaglandins. During the normal postnatal cardiopulmonary transition, the ductus arteriosus typically closes functionally within the first 24 to 72 h of life in healthy term infants. This closure occurs as oxygen levels increase and the levels of prostaglandin E_2_ (PGE_2_) and prostacyclin (PGI_2_) decrease. Anatomical closure of the ductus arteriosus generally occurs by 8 weeks of age in approximately 88% of cases [7,8]. Functional closure is a reversible constriction of the ductus in the first days of life during early postnatal transition to extrauterine life, while anatomical closure due to a complex luminal remodeling and apoptosis, is final, leading to formation of a fibrous ligament called ligamentum arteriosum between aorta and pulmonary artery. Preterm infants are born at various stages of maturation, which can result in delayed closure of the ductus arteriosus, mainly due to a higher sensitivity of the DA to PGE_2_, and NO, and also weaker contractile capacity of the muscle cells [8]. The median time for this closure is 13 days for infants born at 26 to 27 weeks of gestation, while it averages 71 days for those born before 26 weeks [9]. Consequently, extremely preterm infants exhibit increased vulnerability to complications arising from an extended duration of left-to-right shunting.

Very preterm infants, especially ELGANs, experience a wide range of physiological and non-physiological phenomena due to multiorgan immaturity, with jaundice being one of the conditions. Bilirubin metabolism in preterm infants during the early postnatal period is marked by increased production of unconjugated bilirubin, limited hepatic uptake, and challenges in intrahepatic transport, conjugation, and elimination. For instance, at 30 weeks of gestation, the activity of the enzyme uridine diphosphate glucuronosyltransferase (UGT-1A) is only 1/1000 of adult levels, reaching about 1% of adult enzyme activity by term [10]. Low albumin levels in preterm infants can also impair the transport of bilirubin in the plasma, potentially leading to excessively high levels of unconjugated serum bilirubin [11]. This elevated bilirubin level can cross the immature blood–brain barrier, causing bilirubin encephalopathy, which may have irreversible effects. The risk of neurological toxicity decreases as gestational age increases, although the specific bilirubin levels considered toxic have not yet been established, due to the heterogeneity of the neonatal population regarding physiological aspects and associated pathology, which may influence the risk for chronic bilirubin encephalopathy [12,13].

The most common intervention for treating neonatal jaundice and preventing bilirubin encephalopathy is phototherapy (PT). Guidelines for initiating phototherapy are well established in the term and near-term newborns based on hour-specific nomograms for hyperbilirubinemia in the presence or absence of other risk factors [14]. In case of prematurity < 35 weeks of gestation, creating such nomograms is more challenging, as these nomograms should take into account the gestational age and birth weight, along with other risk factors such as acidosis, hypoalbuminemia, asphyxia, hypothermia, much lower total serum bilirubin (TSB) thresholds being used for initiating PT [13,15]. In term infants, guidelines recommend light in the blue-green wavelength range—460–490 nm, at an irradiance of 25–35 µW/cm^2^/nm to at least one surface of the body, administered continuously or intermittently, total duration being led by serial monitoring of TSB concentration [14,16]. The gestational age at which the blood–brain barrier is sufficiently matured is unknown, making preterm infants at greater risk for developing bilirubin-induced brain injury. Considering the potential side effects of phototherapy, such as photo oxidative injury to cell membranes, skin DNA damage, electrolyte imbalances, cardiovascular changes, retinal damage, bronze baby syndrome, etc., PT should be administered as medication [13,15,16]. In ELBW infants without any other risk factors it is recommended an initial irradiance level of 10–30 µW/cm^2^/nm be administered to one body surface area, which should be increased up to 30–49 µW/cm^2^/nm, over the maximum exposed body surface area if additional risk factors are present or the TSB reaches near exchange transfusion thresholds within 2.9 mg/dL of the threshold. PT may be discontinued when TSB levels are 1.5 mg/dL below the phototherapy threshold. In any cases judicious assessment of the need for PT should be performed [15]. Phototherapy is a widely used, non-invasive procedure that addresses neonatal indirect hyperbilirubinemia by converting non-polar, hydrophobic unconjugated bilirubin into hydrophilic bilirubin isomers. These isomers can then be excreted in bile and urine, without requiring conjugation in the liver [17]. Although PT is generally regarded as a relatively safe intervention, it may be associated with some side effects. These can include dehydration, electrolyte imbalances, and even cardiovascular changes. Cardiovascular effects may involve skin blood vessel dilation, increased blood flow to the brain, and reduced blood flow to the splanchnic and renal areas due to the redistribution of blood. Additionally, a decrease in stroke volume can lead to lower cardiac output, influencing the blood flow through the ductus arteriosus, especially noted in preterm infants [18]. Protective chest shielding, if effective, could be a simple, low-cost way to reduce complications. However, previous systematic reviews of two small, randomized trials found inconclusive evidence regarding its effectiveness on the postnatal evolution of the ductus arteriosus [19,20].

The significance of this review is that hemodynamically significant PDA (hsPDA) continues to be a major health issue in very preterm infants, especially in ELGANs, who require phototherapy during a particularly vulnerable transitional postnatal period. This review and meta-analysis aim to examine the available evidence regarding the impact of phototherapy on ductus arteriosus patency and hemodynamics in very preterm infants. Additionally, it aims to determine whether chest shielding is effective in reducing hemodynamic changes.

## 2. Materials and Methods

We adhered to the updated guidelines of the Preferred Reporting Items for Systematic Reviews and Meta-Analyses (PRISMA) 2020 statement, focusing on search strategies, result synthesis, and interpretation (File S1) [21]. The review was registered to PROSPERO international prospective register of systematic reviews, under registration number CRD420251108641 (File S2).

### 2.1. Target Population, Interventions, Outcomes

Our target population consisted of very preterm and extremely preterm infants with a gestational age of 32 weeks or less, who received phototherapy within the first 72 h of life and were evaluated for clinical and/or echocardiographic signs of PDA. The assessment involved the use of a chest shield made of photo-opaque material (aluminum foil with gauze on the left side of the chest), which covered no more than 10% of the body surface area and prevented light penetration. Comparisons were made with non-shielding conditions, where no protection was applied, and sham-shielding, which involved using non-photo-opaque material as a simulated shield, but without preventing light to penetrate through the shield.

In order to evaluate the vasorelaxing effect of light on the ductus arteriosus, we considered as primary outcome the reopening of a previously functionally closed DA or the development of hsPDA during exposure to phototherapy. Both clinical and echocardiographic criteria were considered. Clinically, symptomatic PDA may manifest as a heart murmur, a widened pulse pressure, and a requirement for a fraction of inspired oxygen of 30% or greater.

Echocardiographic diagnosis of PDA—key assessments include:

Ductal characteristics: evaluating the size of the ductal diameter, with a diameter greater than 2 mm considered large in preterm infants. Additionally, a left-to-right shunt across the duct is noted.

1. Signs of pulmonary hyperperfusion: an increased left atrium to aorta (LA/Ao) root ratio greater than 1.4, elevated end-diastolic blood flow in the left pulmonary artery (LPA) exceeding 0.4 m/s, and an enlarged left ventricle end-diastolic diameter.
2. Signs of systemic hypoperfusion: absent or reversed flow in the descending aorta, the ductal steal phenomenon, which presents as low or reversed diastolic flow in the coeliac trunk, superior mesenteric artery, renal arteries, and cerebral arteries.
3. Myocardial function: evaluation of ventricular contractility, left ventricular fractional shortening, ejection fraction, and both systolic and diastolic functions. This structured approach enables accurate diagnosis and management of cardiac conditions in patients [22].

Secondary outcomes, including intraventricular hemorrhage (IVH), persistent or worsening respiratory distress syndrome (RDS), chronic lung disease (CLD), retinopathy of prematurity (ROP), and necrotizing enterocolitis (NEC), were analyzed as the main complications related to prematurity and hsPDA.

### 2.2. Eligibility Criteria

We included in our review only randomized controlled trials (RCTs) published in English language that focused on the target population mentioned above and for which the full text was available. We excluded guidelines, book chapters, systematic or nonsystematic reviews, case reports, case series, observational studies, retrospective studies, and abstracts. Additionally, we excluded studies that involved infants with gestational ages greater than 32 weeks. We also reviewed the reference lists of the articles for any additional relevant studies.

### 2.3. Identification of Studies and Data Collection

We conducted a comprehensive search of the World Wide Web for articles relevant to our selected themes published before June 2025. The literature search was conducted across several databases, including PubMed, EMBASE (Science Direct), Scopus, CINAHL, Google Scholar, and ResearchGate. The keywords used for the general search on Science Direct and CINAHL were as follows: “phototherapy and PDA and preterm or premature,” with an additional filter for “chest shielding.” On PubMed the search terms were as follows: ((((“preterm” [All Fields] OR “preterms” [All Fields] OR (“premature birth” [MeSH Terms] OR (“premature” [All Fields] AND “birth” [All Fields]) OR “premature birth” [All Fields] OR “premature” [All Fields] OR “prematurely” [All Fields] OR “prematures” [All Fields] OR “prematurities” [All Fields] OR “prematurity” [All Fields]) OR (“infant” [MeSH Terms] OR “infant” [All Fields] OR “infants” [All Fields] OR “infant s” [All Fields]) OR (“elgan” [All Fields] OR “elgans” [All Fields])) AND “PDA” [All Fields]) OR (“ductus arteriosus” [MeSH Terms] OR (“ductus” [All Fields] AND “arteriosus” [All Fields]) OR “ductus arteriosus” [All Fields])) AND (“phototherapy” [MeSH Terms] OR “phototherapy” [All Fields] OR “phototherapies” [All Fields]) AND ((“chested” [All Fields] OR “thorax” [MeSH Terms] OR “thorax” [All Fields] OR “chest” [All Fields] OR “chests” [All Fields]) AND (“shield” [All Fields] OR “shielded” [All Fields] OR “shielding” [All Fields] OR “shieldings” [All Fields] OR “shields” [All Fields]))) AND ((1950/1/1:2025/2/12 [pdat]) AND (english [Filter])). Scopus, Google Scholar, and Research Gate, as large databases of academic activity were also searched using the general keywords mentioned.

The selection and data collection were conducted independently by two authors, between 20 November 2024 and 2 June 2025. In cases of disagreement, final decisions were reached through a reasoned discussion in the presence of a third author. The data were organized in an Excel spreadsheet and included the following information: the first author, publication year, journal name, study design, demographic characteristics of the study groups, type of intervention, duration of phototherapy, method of PDA assessment, as well as the primary and secondary outcomes, and the prophylactic and therapeutic strategies used.

### 2.4. Statistical Analysis

We utilized the R packages “meta” and “metasens” of the R statistical software environment, version 4.5.1 for Windows, along with MetaAnalysisOnline [23], to conduct our meta-analysis. Our null hypothesis stated that chest shielding is not more effective than standard care or sham shielding in protecting the ductus arteriosus from the photorelaxation effect of phototherapy. Paule and Mandel’s method was used to quantify heterogeneity between studies by calculating the ԏ^2^, ԏ, I^2^, Q-, and *p*-value. To assure more reliability of these tests for our small dataset, in our interpretation we combined the results of more parameters. As Q test has low power in case of small number of analyzed studies, at this point a *p*-value of less than 0.1 was considered significant, indicating variation in effect size. An I^2^ less than 25% was considered as low heterogeneity, between 25 and 50% as moderate, and over 50% as high heterogeneity.

Prediction intervals were calculated to describe the variation in effect sizes between studies. Data from the primary outcome were used to generate a forest plot, providing a clearer summarized result. Pooled risk ratios (RRs) were used to estimate the summarized effect of the intervention, within 95% confidence intervals (CIs). The random effect estimate was calculated using the Mantel–Haenszel method. Funnel plots and Egger’s test for publication bias testing were performed, but not considered, to avoid misinterpretation, due to their low statistical value in datasets smaller than ten studies.

## 3. Results

**Figure 1:**
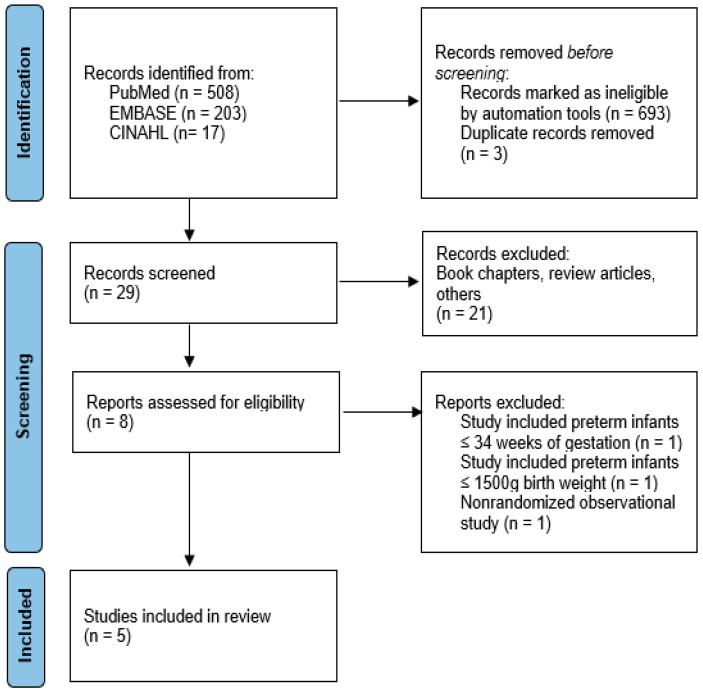
Flow diagram of study identification and selection.

After conducting a general search using our initial sets of keywords, we identified a total of 203 items on EMBASE (via ScienceDirect), 17 on CINAHL, and 508 on PubMed. After applying further filters, we narrowed this down to 8 on PubMed, 23 items in medicine and dentistry subject areas on Science Direct, and 4 articles on CINAHL. After eliminating duplicates (3), excluding book chapters (10), review articles (4), or other articles that did not meet our eligibility criteria (7) and those that included gestational ages >32 weeks or birth weight 1500 g (2), or were nonrandomized observational studies (1), five randomized controlled trials were finally selected to be analyzed, as shown in the flow diagram (Figure 1).

We analyzed data from five randomized controlled studies conducted between 1986 and 2024 [24,25,26,27,28]. A total of 452 infants, with a mean gestational age of 28.04 weeks and a mean birth weight of 1004.8 g, were included in our meta-analysis from these five RCTs. The main characteristics of each study are presented in Table 1, Table 2, Table 3 and Table 4.

Two studies included preterm infants with a GA of less than 29 weeks (Travadi [25] and Mannan [28]). Two other studies (Rosenfeld [24] and Kapoor [26]) focused on infants with a GA of 32 weeks or less. In the Rosenfeld study [24], extremely low birth weight (ELBW) infants, who accounted for 51.35% of the sample, were discussed separately as a subgroup. Additionally, one study (Bozkaya [27]) included infants with a GA of less than 30 weeks. The exclusion criteria for the studies were as follows: in the Rosenfeld study [24], participants were excluded if they died before completing phototherapy (PT); in four studies, participants were excluded if they had congenital heart disease or major congenital anomalies; in the Travadi [25] and Bozkaya [27] studies, participants were excluded if they had Rh isoimmunization or required an exchange transfusion; in the Kapoor study [26], participants with a hemodynamically significant patent ductus arteriosus (hsPDA) before starting PT were excluded. For both the Bozkaya [27] and Mannan [28] studies, individuals who received PT 24 h before or without consent were excluded. Finally, the Mannan study [28] excluded participants who underwent chest shielding after starting PT.

Four studies utilized computer software for randomization, which was conducted by an individual not involved in the study, ensuring proper allocation concealment. In four studies, parental consent was obtained before enrollment and/or randomization [25,26,27,28]. In contrast, the Rosenfeld study [24] only obtained informed consent from the parents of infants in the shielded group. In this study, randomization was performed using a chart after patients were sorted by sex. Notably, Rosenfeld [24] did not implement blinding, whereas all the subsequent studies were blinded [25,26,27,28].

One study (Rosenfeld, 1986 [24]) was conducted before prophylactic antenatal corticosteroids were administered to mothers with imminent premature labor; the other four studies were conducted after the introduction of prophylaxis. Travadi (2006) [25] did not mention whether prophylaxis was given in his study. In the other three studies, antenatal steroid prophylaxis was used in 69.3% of cases in Kapoor’s study [26], 72.28% in Bozkaya’s study [27], and 64.28% in Mannan’s study [28].

In the intervention group, chest shielding was applied to the left side of the chest using aluminum foil covered with gauze or another protective layer. The non-irradiated area did not exceed 10% of the infant’s total body surface area. All five studies employed the same method described by Rubaltelli and Hegyi [30,31]. In each study, the irradiation measurements indicated no exposure under the shielded area. Chest shielding was applied before PT began and was removed only if PT was not in progress. Phototherapy (PT) was initiated after 24 h in all studies. The duration of treatment varied significantly, averaging 46 h in the Travadi study and extending up to 8.4 days in the Rosenfeld study [24]. Each study measured the spectral irradiance of light, which ranged from an average of 20 µW/cm^2^/nm in the Mannan study [28], 22.4 µW/cm^2^/nm in the Kapoor study [26], and 4.77 µW/cm^2^/nm in the Rosenfeld study [24].

**Figure 2:**
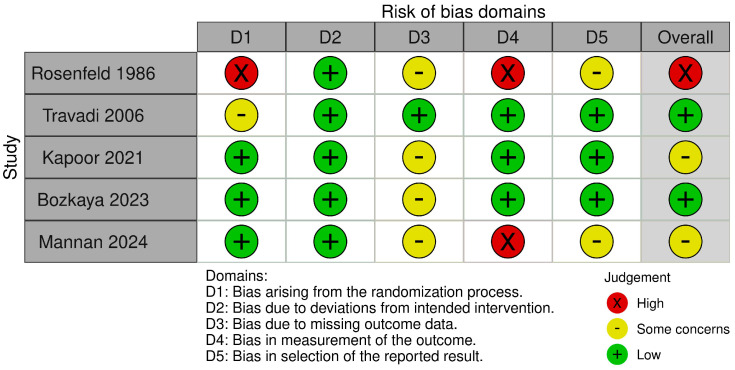
Assessment of the risk of bias between studies [24,25,26,27,28].

PDA assessment was conducted through clinical and/or echocardiographic methods; the studies revealed some variability in both the assessment schedules and the criteria evaluated. The risk of bias was assessed using the ROB 2 tool [32]. We assessed the Rosenfeld study [24] as having a high risk of bias related to randomization and outcome measurement. We also noted additional concerns regarding missing data and the selection of reported results. The Travadi study [25] was assessed as having an overall low risk of bias, although some concerns were raised regarding the randomization process. Both the Kapoor [26] and Bozkaya [27] studies raised some concerns regarding missing outcome data. The Mannan study [28] was assessed as having a high risk in measuring the outcome, along with concerns related to missing data and the selection of reported results. A summary of these assessments is presented in Figure 2.

**Figure 3:**
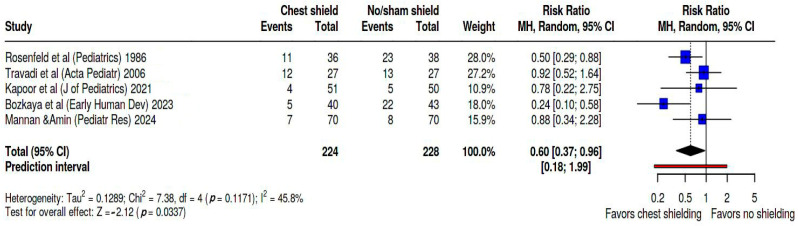
Forest plot representing the pooled RR of PDA occurrence during phototherapy in the studied groups [24,25,26,27,28].

Figure 3 presents the forest plot illustrating the risk ratio (RR) of developing patent ductus arteriosus (PDA) in infants undergoing phototherapy with chest shielding compared to those without shielding or receiving sham shielding. A random effects model was employed for this analysis, and the pooled risk ratio was estimated for the development of PDA among chest-shielded versus non-shielded infants. In our meta-analysis, the studies contributed weights as follows: Rosenfeld’s study [24] accounted for 28%, Travadi’s [25] for 27.2%, Kapoor’s [26] for 10.9%, Bozkaya’s [27] for 18.0%, and Mannan’s [28] for 15.9%. Based on the analysis performed by using a random effects model along with the Mantel–Haenszel method to compare the risk ratios, a statistically significant difference was observed between the two cohorts. The overall risk ratio for PDA development was found to be 0.6, with a 95% confidence interval ranging from 0.37 to 0.96. Heterogeneity was evaluated as moderate, as Tau^2^ = 0.13, Q statistic = 7.38, with a *p* = 0.1 I^2^ = 46%, and prediction interval = 0.18–1.99.

**Figure 4:**
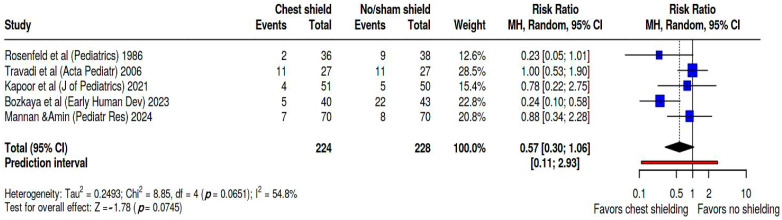
Forest plot representing the pooled RR of hsPDA occurrence during phototherapy in shielded versus non-shielded groups [24,25,26,27,28].

Additionally, we calculated the pooled risk ratio specifically for hemodynamically significant PDA (hsPDA) confirmed by echocardiography, which is displayed in Figure 4, showing a pooled RR of 0.57 (95% CI. 0.3; 1.06, and a wide prediction interval of 0.11; 2.93). Heterogeneity between studies was assessed as significant, with Tau^2^ = 0.25, Q statistic = 8.85 with a *p* = 0.07, and I^2^ = 55%. The studies contributed weights changed as follows: Rosenfeld’s study [24] accounted for 12.6%, Travadi’s [25] for 28.5%, Kapoor’s [26] for 15.4%, Bozkaya’s [27] for 22.8%, and Mannan’s [28] for 20.8%.

Regarding secondary outcomes, IVH was evaluated in 4 studies, ROP in 3 studies, CLD/BPD in 2 studies, NEC in 1 study, and AKI in 1 study. Bozkaya et al. [27] found significant differences between groups in all three secondary outcomes that were assessed. IVH occurred 7.46 (*p* = 0.00) times more in the standard group versus intervention group, while BPD was 1.5 (*p* = 0.001) times more frequent and ROP 2.33 (*p* = 0.006) times. Other studies did not find significant differences in the incidence of secondary outcomes between groups. Kapoor’s study [26] was the only one to assess AKI but found no significant difference between the groups.

**Figure 5:**
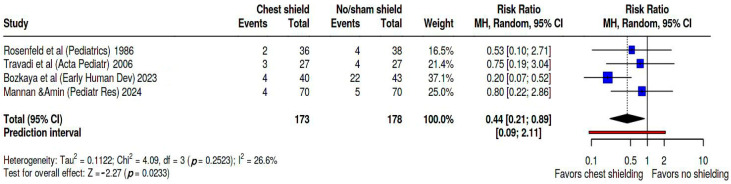
Forest plot representing pooled RR of developing IVH in chest-shielded versus non-shielded groups [24,25,27,28].

We performed a meta-analysis to evaluate the incidence of IVH, the main early complication in this preterm population and the most consistent secondary outcome evaluated among the analyzed studies. Figure 5 shows these findings on forest plot. Pooled RR was 0.44 (95% CI: 0.21; 0.89). Heterogeneity was evaluated as moderate, as Tau^2^ = 0.11, Q statistic = 4.09, with a *p* = 0.25 I^2^ = 27%, and a prediction interval of 0.09; 2.11. In this dataset Bozkaya’s study [27] weighed 37.1%, Mannan study [28] 25%, Travadi study [25] 21.4%, and Rosenfeld study [24] 16.5%. Kapoor’s study [26] did not measure IVH as secondary outcome.

## 4. Discussion

An hsPDA in very preterm and extremely preterm infants significantly impacts overall outcomes due to its association with early and late complications of prematurity. Over time, the management of neonatal intensive care has transitioned from a highly aggressive approach to a more conservative and expectant management strategy. Despite significant progress in understanding the mechanisms behind hemodynamic changes after birth and in developing tools for early diagnosis and point-of-care monitoring, we still lack sufficient knowledge about timing, selection, and methods of treatment. This uncertainty is particularly concerning because therapeutic approaches can also lead to side effects and complications [33,34]. In addition to further research on managing potentially harmful hsPDA, exploring preventive interventions is also beneficial, especially when these interventions are inexpensive and have few or no side effects.

Jaundice is a common condition that occurs during the postnatal transition, affecting more than 80% of preterm infants. While it is well established that preterm infants—particularly those who are very or extremely preterm—are at a higher risk of developing bilirubin encephalopathy, there is a lack of evidence-based guidelines for bilirubin levels that would necessitate treatment based on different gestational ages and birth weights [13]. Neurotoxicity was found to be associated with lower birthweight and gestational age, the presence of cerebral injury, decreased serum albumin concentration, prolonged acidosis, hypothermia, and sepsis. On the other hand, in term and near-term infants there are clear guidelines referring to treatment threshold for indirect hyperbilirubinemia, last updated in 2022 [14]. Establishment of such threshold in preterm infants is more challenging due to the paucity of data referring to this heterogeneous premature population [13]. Chronic bilirubin encephalopathy in preterm infants had been reported at total serum bilirubin levels between as low as 7.5–11.9 mg/dL [15]. This uncertainty is why phototherapy is frequently administered to this population, even at lower serum bilirubin levels. Due to limited data for evidence-based guidelines, there are available consensus-based recommendations. Such a recommendation was published by the group of authors Maisels, Watchko, Bhutani, and Stevenson in 2012, offering suggestions of TSB thresholds for PT and EST for prematures with gestational ages < 28 (0/7)–34 (6/7) [13]. A more detailed consensus-based treatment chart for infants with gestational ages between 22 (+0)–34 (+6) weeks in the first 96 h was developed and published in NeoReviews in 2020 by Pillai et Al [15]. There is not enough evidence whether continuous or intermittent phototherapy has any advantages over the other, although it seems that intermittent PT may be as efficacious as continuous PT. In extremely preterm infants, due to possible side effects, cycled exposure to light of 15 min/h is being studied [16].

In 1961, Furchgott and colleagues observed that exposure to intense light-caused contracted aorta strips from rabbits, cats, and rats to relax reversibly. This finding suggested the existence of an endogenous photosensitive compound in the aortic smooth muscle that, when activated by a specific level of radiation, inhibits contraction. The same photoactivated relaxation was observed in other smooth muscles, such as the stomach and intestinal smooth muscle [35]. A similar phenomenon of photorelaxation was observed by Clyman and Rudolph in vitro, specifically in isolated preterm lamb ductal rings exposed to room light. These preterm rings exhibited a limited response to oxygen-induced contraction, unlike mature vessels. This observation suggests that the walls of premature ducts may be more sensitive to light [36]. In 2003 Andrews et al. found that mouse aorta responded to ultraviolet (UV) light exposure with vasorelaxation, in a reversible manner, regardless of the presence or absence of endothelial NOS (eNOS) deficiency, suggesting that photorelaxation occurs by the involvement of S-nitrosothiols, cGMP, and voltage dependent K^+^ channel activation [37]. Similar findings were published by Batenburg et al. in 2009 in a research involving porcine coronary arteries exposed to light, demonstrating that smooth muscle relaxation is related to the stored S-nitrosothiols, which activate the endothelial intermediate-conductance and small-conductance Ca^2+^-dependent K^+^ channels, leading to subsequent smooth muscle hyperpolarization by activating Na^+^-K^+^ ATP-ase [38]. Subsequent studies revealed a higher incidence of patent ductus arteriosus (PDA) and other hemodynamic changes in preterm infants undergoing phototherapy. This suggests that the intense light radiation may penetrate the thin chest walls of very and extremely preterm infants, directly influencing the physiological closure of the ductus. Various mechanisms have been proposed to explain this, including direct photorelaxation, systemic vasodilation, decreased cardiac output, and the redistribution of blood flow [18,39,40].

Benders et al. (1999) [41] conducted a non-randomized, non-blind observational study to assess the effects of phototherapy on ductal reopening and cardiac output in infants with a gestational age of less than 32 weeks. All infants enrolled in the study had a closed ductus arteriosus (DA) at the time of enrollment. The study found that the ductus reopened in 50% of preterm infants who were exposed to phototherapy [41]. Additionally, Barefield et al. reported an increase in the incidence of patent ductus arteriosus (PDA) among extremely low birth weight (ELBW) infants who were exposed to phototherapy, with rates of 75% compared to 53% of those who were not exposed [42].

Shielding was first utilized by Rubaltelli in 1978 and subsequently by Hegyi in 1981 to protect the hepatic area during phototherapy. This application demonstrated that shielding significantly reduced the effectiveness of phototherapy [30,31].

Some authors reported that there is no link between phototherapy and ductal patency. In 2014, Surmeli-Onay et al. published a prospective observational study examining the effects of phototherapy on the incidence of PDA and its relationship with serum levels of prostaglandin E2 (PGE2) and prostaglandin I2 (PGI2) in preterm infants with a gestational age of less than 34 weeks. Prostaglandins, particularly PGE2, are known as potent vasodilators that play a crucial role in maintaining ductal patency. A total of 44 infants were enrolled, divided into non-PDA and PDA groups at the beginning of the study, based on echocardiographic findings. In the non-PDA group ductal reopening was observed in 3 preterm infants (14.3%), while ductal closure in PDA group was present in 4 cases (17.3%) during phototherapy; leading to the conclusion that phototherapy did not affect either ductal patency, or the variations of PGE_2_ [43].

The concept of chest shielding aims to minimize the direct impact of PT on the vascular bed, particularly the ductus arteriosus. In all studies, the chest shield covers about 10% of the total body surface area and is made of photo-opaque material, blocking light from passing through the chest wall.

Previous systematic reviews and meta-analyses have yielded contradictory results when analyzing two small, randomized trials. The Cochrane systematic review conducted by Bhola et al. [19] in 2015 and a meta-analysis performed by Mannan et al. [20] in 2017 included the same two RCTs: those of Rosenfeld et al. [24] and Travadi et al. [25]. These two studies demonstrated high heterogeneity during the neonatal era, with varying therapeutic approaches, demographic characteristics of the target population, differences in randomization, modes of assessment, and blinded versus non-blinded design. The conclusion of the analyses was conflicting, showing insufficient evidence to assess the safety and efficacy of chest shielding during phototherapy [19,20].

In our review, we included three additional blinded, randomized controlled trials conducted over the last five years, all with the same goal but differing study designs.

The study of Rosenfeld is the first on the timeline and is the most rudimentary in design. It was conducted in 1986, before the antenatal steroid era, and included 74 preterm infants with GA between 26 and 32 weeks. The study is a non-blinded RCT comparing clinically evident PDA in preterm infants who received PT, divided into a chest-shielded group and a non-shielded group. They also included a subgroup of extremely low birth weight (ELBW) infants, representing 50% of the total number assessed. One potential source of bias was that written consent was obtained only from the parents of the shielded group after the randomization process had been completed. The inclusion and exclusion criteria are not clearly defined. Prophylactic phototherapy was used in all cases, with a mean irradiance of 4.77 µW/cm^2^/nm, for a mean duration of 8.3 days, without significant differences between groups regarding TSB levels or need for exchange transfusion. PDA assessment was performed clinically, while echocardiography was performed only on infants showing clinical signs of PDA; silent PDA remained undetected. The study found a significantly lower incidence of PDA in the shielded group (*p* = 0.009), with these findings primarily due to significance in the ELBW group (*p* = 0.007); no significant differences were observed in the more mature group. The main risk of bias was in the measurement of the outcome, as the actual incidence of PDA could not be accurately defined due to the limitations of echocardiographic evaluation [24].

In 2006, Travadi et al. [25] conducted a blinded randomized controlled trial (RCT) following the worldwide implementation of antenatal steroid prophylaxis. The study had well-defined inclusion and exclusion criteria. A total of 54 infants, all with a gestational age of less than 29 weeks, were enrolled and randomized in a 1:1 ratio. The infants were divided into two groups as follows: 13/13 infants were under 27 weeks of gestation, and 14/14 infants were between 27 and 28 weeks of gestation. Randomization was conducted using a computer-generated random sequence, and the allocation was performed by sequentially selecting opaque, numbered envelopes. Phototherapy was started at a TSB level of 107/104 mmol/L, at a mean postnatal age of 31/27 h, for a mean duration of 46 h in each group. Echocardiographic evaluation was conducted in accordance with international guidelines and was blinded to both the examiner and the study group allocation. Both baseline and post-interventional assessments were made. The study found that chest shielding did not protect against the prevalence or severity of patent ductus arteriosus (PDA), although early administration of indomethacin in 40.7% of cases may have influenced the results [25].

Kapoor et al. conducted their blinded open-label RCT in 2020, enrolling 101 very preterm infants with GA ≤32 weeks who needed PT within 72 h of birth [26]. Randomization was performed using a web-based random number generator by a consultant who had no role in trial enrollment. Echocardiographic assessments were conducted in accordance with international Point-of-Care Ultrasound (POCUS) guidelines [29], with additional data included to enhance the evaluation of the PDA. The initial echocardiographic assessment was performed within 12 to 24 h after birth, or whenever PT was initiated. They used Maisels nomogram for preterm infants less than 35 weeks gestation, from 2012 [13], to indicate phototherapy. PT was initiated at 9.7/10.2 mg/dL TSB levels, at a mean postnatal age of 44.2/45.3 h, and lasted for about 72 h, without significant difference between the groups. Subsequent assessments occurred every 24 h or more frequently if clinical signs suggested the presence of a PDA. Hemodynamically significant PDA was present in about 10% of both groups, with no significant differences between them, and was monitored until closure. This study utilized additional echocardiographic parameters to describe PDA and included blinding and randomization, which reduced bias compared to Rosenfeld’s study. The source of bias might be that all infants with hsPDA were required to be treated with paracetamol, leading to 100% ductal closure after a six-day course. Another limitation of the study is that the enrolled newborns had a mean GA of 30.1 ± 1.5/1.6 weeks; additionally, the ELGAN and ELBW categories were not studied separately. As secondary outcome they measured the effect of PT on echocardiographic parameters of the hsPDA. Although no overall significant differences were found between the groups, the echocardiographic assessment showed significant differences (*p* = 0.03) between LA/Ao ratio during PT, and slightly larger ductal size (1.8 ± 0.2 vs. 2.4 ± 0.9, *p* = 0.2) in hsPDA infants, showing a possible more vasorelaxation in the non-shielded group. They did not assess for other morbidities such as IVH, NEC, CHD, or ROP, except for the presence of acute kidney injury as secondary outcome, without significant difference between groups [26].

Bozkaya et al. (2023) [27] studied the effect of PT and the role of chest shielding protection on the diameter of the ductus arteriosus in extremely premature infants in a blinded RCT. They enrolled 83 preterm infants with BW ≤ 1000 g and GA ≤ 30 weeks of gestation. Randomization was conducted using sequential numbers generated at the NICU’s computer center by a neutral person who was not part of the study team, after the PT indication was made. PT indication followed the American Academy of Pediatrics’ guideline from 2009 and did not show any differences regarding duration between groups. Echocardiography was performed before starting PT and again 2 h after PT was discontinued. They found that ductal diameter and LA/Ao root ratio increased significantly in the control group after PT, even though the baseline DA size was similar before starting PT. The need for PDA treatment also showed significantly higher odds in the non-shielded group compared to the shielded group, concluding that PT increased the incidence of hsPDA, and chest shielding may help protect against DA during PT in ELGANS. The significance of this study lies in the similarity and homogeneity of the groups studied, including GA (27 ± 2/27 ± 1.5 weeks), BW (860 ± 165/836 ± 164 g), mode of delivery, postnatal cardio-respiratory adaptation, CRIB scores, and duration of phototherapy. The study plan was clearly defined and took baseline variables into account, primarily monitoring some outcomes and secondary ones in the same manner. The only potential bias could arise from some missing outcome data [27].

The double-blinded, randomized, placebo-controlled trial conducted by Mannan and Amin in 2024 enrolled 160 infants (80 per group) with GA < 29 weeks, with 70/70 individuals analyzed. Both groups were similar in GA (26.6 ± 1.9/26.8 ± 2.1), BW (853 ± 228/891 ± 239 g), antenatal steroids, mode of delivery, Apgar scores, CRIB II score, RDS requiring surfactant, sepsis, exposure to inotropes, indomethacin for IVH prevention, transfusions, timing and duration of PT, initial and peak total serum Bi levels, fluid intake, and days of mechanical ventilation. The study identifies several potential biases. The echocardiographic assessment of the patent ductus arteriosus (PDA) was performed before the initiation of pharmacological treatment (PT) in only 50 infants. Furthermore, the first examination took place sometime between the first 24 h after starting PT and up to 3 days after stopping PT. Post-intervention reexamination also varies; they reexamined infants diagnosed with hsPDA within 24 h after the onset of symptomatic PDA and only within 72 h after discontinuing PT in cases that did not develop symptomatic PDA. A large percentage of infants underwent surgical ligation due to failure of medical treatment or contraindications for indomethacin. The study found no significant differences in symptomatic PDA, the need for surgical ligation, or secondary outcomes, including the incidence of necrotizing enterocolitis, bronchopulmonary dysplasia, retinopathy of prematurity, or intraventricular hemorrhage. The authors concluded that chest shielding had no impact on either the primary or secondary outcomes for the group of infants studied. However, they recognized that a potential limitation of the study was the use of indomethacin as a prophylactic treatment for IVH in most cases, which may have affected the process of ductus arteriosus closure. The strength of this study is that the target population is very well established and compact [28].

The seemingly contradictory results of these studies are partly due to differences in patient demographics, antenatal steroid exposure, indomethacin prophylaxis, study design, and criteria of hsPDA. The observation that chest shielding does not have uniform efficiency across all patients is highlighted by the predictive interval, which serves as a measure of variability. This indicates that the effectiveness of this intervention differs among individuals. Nevertheless, the data also suggest that it is most beneficial for the most vulnerable patients. According to the analyzed studies, chest shielding with aluminum foil seems to be a harmless, low-cost intervention that does not alter the efficacy of phototherapy [24,25,26,27,28,44].

Limitations of this review also consist in the small number of available RCTs with relatively small sample sizes, with heterogeneity between them regarding study design, target population, diagnostic criteria for hsPDA, and evaluation of primary and secondary outcomes. Limitation of the review also consist in using exclusively data available in published full text studies, without extension to other languages than English. The available data are of low statistical quality and require caution in our final conclusions. Furthermore, better designed multicenter randomized studies focused on the ELGAN category—the most vulnerable premature population in means of immaturity, risk factors, and long-term morbidity—and use of standardized echocardiographic evaluation may provide a more definitive answer regarding effects of PT on ductal closure and cost-effectiveness of a proper chest shield.

## 5. Conclusions

The meta-analysis of data from these five trials suggests a possible moderate protective effect of the chest shield against the development of PDA during phototherapy, with a relative risk of 0.6, indicating a slight favorable effect of chest shielding. Although diminishing efficiency of phototherapy was not noted in chest-shielded patients, the small number of studies and the significant heterogeneity between them require caution in recommending chest shielding for preventing development of hsPDA in exposed infants. Our findings suggest that future RCTs should have a more strategic design with a more uniform target population, and a more standardized assessment tool, considering the significant physiological differences among preterm infants of different gestational ages.

## Figures and Tables

**Table 1:** Characteristics of studies included in the meta-analysis regarding study design.

Author/ Year of Publication/ Country	Type of Study	Inclusion Criteria	Exclusion Criteria	A: Intervention Group Nr	B: Control Group Nr	Total NR	GA Weeks (Mean)	BW Grams (Mean)
Rosenfeld et al., 1986 USA [24]	Non-blinded RCT	≤32 wks, and 1500 g, PT	Death before completion of PT	Chest shield 36	No shield 38	74	A: 26–32 (29.1) B: 26–32 (29.6)	A: 539–1430 (1033) B: 600–1500 (1037)
Travadi et al., 2006 Australia [25]	Blinded RCT	<29 wks requiring PT	CHD, major anomalies, Rh isoimmunization hydrops fetalis, EST	Chest shield 27	No shield 27	54	A: 25–28 (27) B: 25–28 (27)	A: 710–1165 (945) B: 705–1160 (890)
Kapoor et al., 2021 India [26]	Blinded open-label RCT	≤32 wks, admitted to NICU within 24 h	hsPDA before PT, major anomalies, death within 12 h of admission	Chest shield 51	No shield 50	101	A: 30.1 ± 1.5 B: 30.1 ± 1.6	A: 1281 ± 259 B: 1422 ± 307
Bozkaya et al., 2023 Turkey [27]	Blinded RCT	≤30 wks and 1000 g treated with PT after 24 h	CHD, major anomalies, EST, Rh isoimmunization PT before 24 h, inotropes within 72 h	Chest shield 40	No shield 43	83	A: 27 ± 1.5 B: 27 ± 2	A: 836 ± 164 B: 860 ± 165
Mannan and Amin 2024 USA [28]	Double-blinded RCT	<29 wks or 1000 g admitted to NICU within 24 h who needed PT	CHD, moribund condition, PT before consent, PHT on NO, chest shield after initiation of PT	Chest shield 70	Sham shield 70	140	A: 26.6 ± 1.9 B: 26.8 ± 2.1	A: 853 ± 228 B: 891 ± 239

Abbreviations: RCT—randomized controlled trial; GA—gestational age; BW—birth weight; CHD—congenital heart disease; PT—phototherapy; wks—weeks; NICU—neonatal intensive care unit; PDA—patent ductus arteriosus; hsPDA—hemodynamically significant PDA; PHT—pulmonary hypertension; NO—nitric oxide.

**Table 2:** Characteristics of studies included in the meta-analysis regarding phototherapy.

Author/ Year of Publication/ Country	Protocol for PT	PT Device/ Irradiance	TSB Threshold for Starting PT	PT Timing	Duration of PT Mean
Rosenfeld et al., 1986 USA [24]	Prophylactic to all infants <32 weeks of gestation	Air Shields model PTU 78-1/ irradiance > 4.0 µW/cm^2^/nm (mean 4.77)	Not reported	First 24 h of life	48–360 h (201.6 h)
Travadi et al., 2006 Australia [25]	Unit’s own protocol	Medela AG, Switzerland, Hill-Rom Air Shields, Hatboro, PA, USA/ irradiance 3–9 µW/cm^2^/nm	105 mmol/L (6.2 mg/dL)	29 h of mean postnatal age	14–174 h (46 h)
Kapoor et al., 2021 India [26]	2012 Maisels guideline for preterm infants less than 35 weeks of gestation	LED/CFL bulbs/ irradiance 22.4 ± 6.0 µW/cm^2^/nm	9.9 ± 1.4 mg/dL	44.8 h of mean postnatal age	48–96 h (71.7 h)
Bozkaya et al., 2023 Turkey [27]	2009 AAP guidelines for hyperbilirubinemia treatment	Blue Angel: Ertunç Özcan Company Ankara/Turkey	Not reported	After 24 h of life	24–96 h (48 h)
Mannan and Amin 2024 USA [28]	Unit’s own protocol	Natus neo-BLUE light-emitting diode lights/irradiance > 20 µW/cm^2^/nm	Initial TSB not reported. Peak TSB: 7.6 ± 0.7 mg/dL	74.4 h of mean postnatal age	21–288 h (59.15 h)

Abbreviations: PT—phototherapy; TSB—total serum bilirubin; AAP—American Academy of Pediatrics.

**Table 3:** Characteristics of studies included in the meta-analysis regarding PDA assessment, outcome, and treatment.

Author/ Year of Publication/ Country	PDA Clinical Diagnosis	hsPDA Echocardiographic Criteria	PDA Occurrence Based on Clinical Assessment	hsPDA Occurrence Based on Echocardiograpic Assessment	hsPDA Medical Treatment	hsPDA Surgical Ligation
Rosenfeld et al., 1986 USA [24]	Appearance of cardiac murmur suggestive for PDA	LA/Ao root > 1.22	Group A: 11/36 Group B: 23/38	Group A: 2/36 Group B: 9/38	Indomethacin in 1 case of Group B	Group A: 0 Group B: 1
Travadi et al., 2006 Australia [25]	Not reported separately	LA/Ao > 1.4 or ductal diameter > 1.5 mm with left-to-right shunt	NA	Group A: 12/27 Group B: 13/27	Indomethacin in 11 cases in both groups	No
Kapoor et al., 2021 India [26]	Not reported separately	* Ductal diameter ≥ 1.4 mm, plus one of the following: LA/Ao ≥ 1.4, LPA end diastolic flow ≥ 0.2 mL/s, mean flow ≥ 0.4 mL/s reversed diastolic flow in the Ao	NA	Group A: 4/51 Group B: 5/50	Paracetamol in all cases	No
Bozkaya et al., 2023 Turkey [27]	Not reported separately	LA/Ao > 1.5/ductal diameter > 1.5 mm with left-to-right shunt	NA	Group A: 5/40 Group B: 22/43	In all cases, (No data about the substance used)	Group A: 2 Group B: 11
Mannan and Amin 2024 USA [28]	Systolic heart murmur with 2 of the following signs: (1) widened pulse pressure; (2) metabolic acidosis (3) FiO2 ≥ 30%.	Ductal diameter, LA/Ao ratio LA volume	Overall 20/140	Group A: 7/70 Group B: 8/70	Indomethacin in all cases	Group A: 6 Group B: 6

Abbreviations: PDA—patent ductus arteriosus; hsPDA—hemodynamically significant PDA; LA—left atrium; Ao—aorta; LPA—left pulmonary artery; FiO2—fraction of inspired oxygen; A: intervention group, B: control group, *: Based on POCUS guideline of ESPNIC [29].

**Table 4:** Characteristics of included studies regarding the assessment of primary and secondary outcomes.

Author/ Year of Publication/ Country	PDA Assessment Schedule	Primary Outcome	Secondary Outcome	Antenatal Steroid Prophylaxis	IVH Prophylaxis with Indomethacin	Stay in the Hospital
Rosenfeld et al., 1986 USA [24]	Clinical criteria/ Echocardiography only for those with a cardiac murmur	Incidence of PDA, hsPDA *p* < 0.05	IVH: *p* > 0.05 ROP: *p* > 0.05	No	No	Shielded patients had shorter stay
Travadi et al., 2006 Australia [25]	Baseline echocardiography and after 48 h of PT	Incidence of PDA, hsPDA *p* > 0.05	IVH: *p* > 0.05	No data	No	No significant difference
Kapoor et al., 2021 India [26]	Baseline echocardiography before PT and at every 24 h, or if clinically required	Incidence of hsPDA *p* > 0.05	PT effect severity of hsPDA (based on echo parameters): overall *p* > 0.05. AKI: *p* > 0.05	>60% both groups *p* > 0.05	No	Not measured
Bozkaya et al., 2023 Turkey [27]	Baseline echocardiography and 2 h after PT	Incidence of hsPDA *p* < 0.05	IVH: *p* < 0.05 BPD: *p* < 0.05 ROP: *p* < 0.05 In favor for shielding	>70% both groups *p* > 0.05	No	No significant difference
Mannan and Amin 2024 USA [28]	Clinical and echo criteria Baseline echocardiography when available, 24 h after hsPDA diagnosis/within 72 h after PT	Incidence of hsPDA *p* > 0.05	IVH: *p* > 0.05 NEC: *p* > 0.05 CLD: *p* > 0.05 ROP: *p* > 0.05 Surgical ligation: *p* > 0.05	>60% both groups *p* > 0.05	Yes	Not measured

Abbreviations: PDA—patent ductus arteriosus; hsPDA—hemodynamically significant PDA; PT—phototherapy; IVH—intraventricular hemorrhage; ROP—retinopathy of prematurity; NEC—necrotizing enterocolitis; CLD—chronic lung disease; AKI—acute kidney insufficiency; BPD—bronchopulmonary dysplasia.

## Data Availability

Not applicable.

## Supplementary Materials

The following supporting information can be downloaded at https://www.mdpi.com/article/10.3390/biomedicines13102567/s1, File S1: PRISMA checklist; File S2: PROSPERO registration card.

## Abbreviations

The following abbreviations are used in this manuscript:

DA	Ductus arteriosus
PDA	Patent ductus arteriosus
hsPDA	Hemodynamically significant PDA
ELGAN	Extremely low gestational age
ELBW	Extremely low birth weight
PGE2	Prostaglandin E2
PGI2	Prostacyclin
UGT-1A	Enzyme uridine diphosphate glucuronosyltransferase
PT	Phototherapy
TSB	Total serum bilirubin
PRISMA	Preferred Reporting Items for Systematic Reviews and Meta-Analyses
LA	Left atrium
Ao	Aorta
AKI	Acute kidney insufficiency
LPA	Left pulmonary artery
LV	Left ventricle
IVH	Intraventricular hemorrhage
RDS	Respiratory distress syndrome
CHD	Congenital heart disease
CLD	Chronic lung disease
PHT	Pulmonary hypertension
NO	Nitric oxide
NEC	Necrotizing enterocolitis
BPD	Bronchopulmonary dysplasia
ROP	Retinopathy of prematurity
RCT	Randomized controlled trials
MeSH	Medical Subject Headings
RR	Risk ratio
CI	Confidence interval
PI	Prediction interval
POCUS	Point-of-Care Ultrasound
ESPNIC	European Society of Pediatric and Neonatal Intensive Care
AAP	American Academy of Pediatrics
eNOS	Endothelial nitric oxide synthase
ATP	Adenosine triphosphate
