# Supplementary material for: Does Phototherapy Affect Ductus Arteriosus Closure in Preterm Infants ≤32 Weeks of Gestation, and Can We Influence This Through Chest Shielding? Review of the Literature and a Meta-Analysis
Source: Biomedicines. 2025 Oct 21;13(10):2567. doi: 10.3390/biomedicines13102567 (PMC12562127; doi:10.3390/biomedicines13102567)
Supplement: Supplementary file 1 [file biomedicines-13-02567-s001.zip › File S2-PROSPERO registration card.pdf]

NIHR | National Institute for Health and Care Research

PROSPERO
International prospective register of systematic reviews

Does Phototherapy Affect Ductus Arteriosus Closure in Preterm Infants ≤32 Weeks of Gestation, and Can We Influence This Through Chest Shielding? Review of the Literature and a Me-ta-Analysis

Marta Simon, Zsuzsanna Gall, Monika Rusneac, Amalia Fagarasan, Raluca Marian, Madalina Anciuc Crauciuc, Andreea Racean, Andrea Noemi Toth, Manuela Cucerea

Citation
Marta Simon, Zsuzsanna Gall, Monika Rusneac, Amalia Fagarasan, Raluca Marian, Madalina Anciuc Crauciuc, Andreea Racean, Andrea Noemi Toth, Manuela Cucerea. Does Phototherapy Affect Ductus Arteriosus Closure in Preterm Infants ≤32 Weeks of Gestation, and Can We Influence This Through Chest Shielding? Review of the Literature and a Me-ta-Analysis. PROSPERO 2025 CRD420251108641. Available from https://www.crd.york.ac.uk/PROSPERO/view/CRD420251108641.

REVIEW TITLE AND BASIC DETAILS

Review title
Does Phototherapy Affect Ductus Arteriosus Closure in Preterm Infants ≤32 Weeks of Gestation, and Can We Influence This Through Chest Shielding? Review of the Literature and a Me-ta-Analysis

Condition or domain being studied
evolution of patent ductus arteriosus in preterm infants exposed to phototherapy and the protective role of chest shielding

Rationale for the review
PDA and especially hs PDA is a cardiac condition with possible early and late complications. High irradiation during phototherapy for jaundice may have an influence on preterm hemodynamics. Protective procedures such as chest shielding with photo-opaque material may prevent the development of hsPDA in these infants

Review objectives
to evaluate the affect of PT on ductal reopening and the benefits of chest shielding in prevent it

Keywords
Prematurity; Patent ductus arteriosus; Phototherapy; postnatal transition; chest shielding

Country
Romania

ELIGIBILITY CRITERIA

Population

Included
very preterm and extremely preterm infants who underwent phototherapy in the first 72 hs of life with or without chest shielding

Excluded
preterm infants with GA>32 weeks, phototherapy later in life, pathologic jaundice, CHD, ductal dependent CHD

Intervention(s) or exposure(s)

Included
chest shielding during exposure to phototherapy

Excluded
chest shielding after PT

Comparator(s) or control(s)

Included
no shielding or sham shielding during exposure to PT

Excluded
CHD, Rh isoimmunisation, PT later in life

Study design
Only randomized study types will be included.

Included
randomized controlled trials, full text available, English language

Excluded
non randomized studies, book chapters, case reports, observational studies, chest shielding not applied

Context
any RCT that meet the eligibility criteria

TIMELINE OF THE REVIEW

Date of first submission to PROSPERO
21 July 2025

Review timeline
Start date: 20 November 2024. End date: 18 August 2025.

Date of registration in PROSPERO
21 July 2025

AVAILABILITY OF FULL PROTOCOL

Availability of full protocol

A full protocol has been written but is not available because:

we follow the PRISMA protocol that is available

SEARCHING AND SCREENING

Search for unpublished studies

Only published studies will be sought.

Main bibliographic databases that will be searched

The main databases to be searched are *CINAHL - Cumulative Index to Nursing and Allied Health Literature*, *Embase.com*, *MEDLINE*, *PubMed* and *Scopus*.

Search language restrictions

The review will only include studies published in English.

Search date restrictions

There are no search date restrictions.

Other methods of identifying studies

Other studies will be identified by: *reference list checking* and *searching conference proceedings*.

Link to search strategy

A full search strategy is available in the full protocol as described in the *Availability of full protocol* section

Selection process

Studies will be screened independently by at least two people (or person/machine combination) with a process to resolve differences.

Other relevant information about searching and screening

checking reference list

DATA COLLECTION PROCESS

Data extraction from published articles and reports

Data will be extracted independently by at least two people (or person/machine combination) with a process to resolve differences.

Authors will not be contacted for further information.

Study risk of bias or quality assessment

Risk of bias will be assessed using: *Cochrane RoB-2*

Data will be assessed independently by at least two people (or person/machine combination) with a process to resolve differences.

Additional information will **not** be sought from study investigators if required information is unclear or unavailable in the study publications/reports.

Reporting bias assessment

Rob2

Certainty assessment

Certainty of findings will not be assessed

OUTCOMES TO BE ANALYSED

Main outcomes

primary outcomes like PDA and hsPDA

Additional outcomes

secondary outcomes that are mesured in most studies

PLANNED DATA SYNTHESIS

Strategy for data synthesis

we assess heterogenetity among studies using Paul-Mandel method , calculating the tau2, tau, Q-value, H-value, I2, and p-value. We will calculate prediction intervals also. Forest plots will be generated to describe summarized result. publication bias is assessed by generating funnel plots and performing Egger's test. R packages meta and metasens of R statistical software environment along with metaanalysesonline were utilized to conduct the meta-analysis.

CURRENT REVIEW STAGE

Stage of the review at this submission 1 change

Review stage	Started	Completed
Pilot work	✓	✓
Formal searching/study identification	✓	✓
Screening search results against inclusion criteria	✓	✓
Data extraction or receipt of IPD	✓	✓
Risk of bias/quality assessment	✓	✓
Data synthesis	✓	✓

Review status

The review is completed.

Publication of review results

Results of the review will be published in English.

Journal publication

Not yet published in a journal but will be in future.

REVIEW AFFILIATION, FUNDING AND PEER REVIEW

Review team members

Dr Marta Simon (review guarantor and contact) ORCID: 0000-0003-1264-5917. George Emil Palade University of Medicine, Pharmacy, Science and Technology of Targu Mures. Romania.

No conflict of interest declared.

Dr Zsuzsanna Gall. George Emil Palade University of Medicine Pharmacy Science and Technology of Targu Mures. Romania.

No conflict of interest declared.

Dr Monika Rusneac. 2 Department of Neonatology, Targu Mures County Emergency Clinical Hospital. Romania.

No conflict of interest declared.

Dr Amalia Fagarasan. George Emil Palade University of Medicine Pharmacy Science and Technology of Targu Mures. Romania.

No conflict of interest declared.

Dr Raluca Marian. George Emil Palade University of Medicine Pharmacy Science and Technology of Targu Mures. Romania.

No conflict of interest declared.

Dr Madalina Anciuc Crauciuc. George Emil Palade University of Medicine Pharmacy Science and Technology of Targu Mures. Romania.

No conflict of interest declared.

Dr Andreea Racean. George Emil Palade University of Medicine Pharmacy Science and Technology of Targu Mures. Romania.

No conflict of interest declared.

Dr Andrea Noemi Toth. George Emil Palade University of Medicine Pharmacy Science and Technology of Targu Mures. Romania.

No conflict of interest declared.

Associate Professor Manuela Cucerea. George Emil Palade University of Medicine Pharmacy Science and Technology of Targu Mures. Romania.

No conflict of interest declared.

Named contact

Dr Marta Simon (marta.simon@umfst.ro). ORCID: 0000-0003-1264-5917. George Emil Palade University of Medicine, Pharmacy, Science and Technology of Targu Mures. Romania.

Review affiliation

George Emil Palade University of Medicine Pharmacy Science and Technology of Targu Mures

Funding source

Grant number
10126/3, 17.12.2020

Additional commercial funding information
This research was funded by the George Emil Palade University of Medicine, Pharmacy, Sciences and Technology of Targu Mures

Peer review
There has been no peer review of this planned review.

ADDITIONAL INFORMATION

Review conflict of interest
Declared individual interests are recorded under team member details. This review is funded by a commercial organisation.. No additional interests are recorded for this review.

Medical Subject Headings
Ductus Arteriosus; Ductus Arteriosus, Patent; Humans; Infant; Infant, Newborn; Infant, Premature; Phototherapy

Revision note 1 change
The search and meta-analysis had been completed and is under review for publication

SIMILAR REVIEWS

Check for similar records already in PROSPERO
PROSPERO identified a number of existing PROSPERO records that were similar to this one (last check made on 20 July 2025). These are shown below along with the reasons given by that the review team for the reviews being different and/or proceeding.

- Efficacy of using a shielding technique during phototherapy on prevention of incidence of significant patent ductus arteriosus in premature infants: A systematic review and meta-analysis [published 8 September 2024] [CRD42024584558]. The review was acknowledged as **similar** but the authors opted to continue because *there are differences in population*, *there are differences in intervention or comparator*, *the review looks at additional or different outcomes*, *the review will be more up to date*
- Stress shielding in reverse total shoulder arthroplasty and its effect on clinical outcome [published 9 September 2024] [CRD42024585085]. The review was judged **not to be similar**
- Enteral feeding in preterm infants during treatment with cyclooxygenase inhibitor for patent ductus arteriosus: A systematic review and meta-analysis [published 19 June 2023] [CRD42023374092]. The review was judged **not to be similar**

PROSPERO version history 1 change

- Version 1.1, published 15 Sep 2025
- Version 1.0, published 21 Jul 2025

Disclaimer
The content of this record displays the information provided by the review team. PROSPERO does not peer review registration records or endorse their content.

PROSPERO accepts and posts the information provided in good faith; responsibility for record content rests with the review team. The guarantor for this record has affirmed that the information provided is truthful and that they understand that deliberate provision of inaccurate information may be construed as scientific misconduct.

PROSPERO does not accept any liability for the content provided in this record or for its use. Readers use the information provided in this record at their own risk.

Any enquiries about the record should be referred to the named review contact
